# Poly(Vinyl Alcohol)–Carbon Nanotube Self−Adhesive Hydrogels for Wearable Strain Sensors

**DOI:** 10.3390/polym17162249

**Published:** 2025-08-20

**Authors:** Guofan Zeng, Nuozhou Yi, Qiaohang Guo, Fei Han, Mingcen Weng

**Affiliations:** 1 Department of Physical Education, Fujian University of Technology, Fuzhou 350118, China; 2 Institute of Biology and Chemistry, Fujian University of Technology, Fuzhou 350118, China; 3 School of Materials Science and Engineering, Fujian University of Technology, Fuzhou 350118, China; 4 The Key Laboratory of Biomedical Information Engineering of Ministry of Education, School of Life Science and Technology, Xi’an Jiaotong University, Xi’an 710049, China; 5 Bioinspired Engineering and Biomechanics Center (BEBC), Xi’an Jiaotong University, Xi’an 710049, China

**Keywords:** wearable electronics, PVA, CNT, hydrogel, self−adhesive, strain sensor

## Abstract

Wearable conductive hydrogel sensors, which are highly convenient, have attracted attention for their great potential in human motion monitoring and smart healthcare. However, the self−adhesive properties, sensing performance, and stability of traditional hydrogels are not ideal, which seriously hinders their use in monitoring and diagnosing joints throughout the human body. Here, CaCl_2_ is used to crosslink PVA to improve its self−adhesive properties, and it is then combined with a CNT conductive network. Next, a cyclic freeze–thaw strategy is utilized to fabricate a wearable PVA−Ca−CNT hydrogel with excellent self-adhesive properties and stability. PVA−Ca−CNT hydrogels can adhere to various substrates, with a maximum self-adhesion strength of 398 kPa and a unit adhesion energy of as high as 305 μJ cm^−2^. Furthermore, the CNT three−dimensional network enhances the tensile strength to 110 kPa, with almost no hysteresis. Based on resistance changes, PVA−Ca−CNT hydrogel exhibits a sensitivity of up to 11.11 as a strain sensor as well as a response to strain stimuli within 180 ms. When PVA−Ca−CNT hydrogel is adhered to the surface of human skin, it operates as a sensor for monitoring human movement. Not only can it accurately monitor the movement positions of major joints in the human body, it can also accurately identify tiny movements of the fingers and be used as a finger Morse code output device, which demonstrates the enormous potential of human motion monitoring systems based on self−adhesive hydrogel sensors in practical applications.

## 1. Introduction

With the growth of smart materials and intelligent healthcare, flexible wearable sensors have gotten more attention due to their unique convenience [1,2,3,4]. Hydrogels composed of three−dimensional hydrophilic networks have become popular substrate materials to prepare flexible wearable sensors due to their excellent biocompatibility and biomimetic properties [5,6]. Pure hydrogel materials lack conductivity and are therefore unsuitable as substrates for wearable devices [7,8]. Therefore, researchers add conductive fillers to increase their conductivity [9]. Researchers generally use metal−based nanomaterials to enhance the mechanical properties and conductivity of hydrogels, including noble metals (e.g., Ag and Au) or metal oxides (e.g., Fe_3_O_4_ and TiO_2_) [10,11]. However, the challenges of mechanical tunability and high cost of hydrogels remain unresolved. Lai et al. introduced Al ions into hydrogels, increasing strain from 400% to 1400%, but significant hysteresis occurred, making stable use unsuitable [12]. To improve stability, Zeng et al. mixed CNT with PVA, which not only improved conductivity but also increased the working stability time of the hydrogel to over 12,000 s [13]. However, hydrogel generally requires adhesive tape for fixation during use, lacking self−adhesive properties. Therefore, it is of great significance to develop a hydrogel with self−adhesive properties and ideal stability as a substrate for flexible wearable devices [14].

PVA, as a flexible material with excellent biocompatibility, is widely used in biomedicine, flexible sensing, and human–computer interaction fields [15,16]. Manchi et al. designed a flexible and sensitive single−electrode triboelectric nanogenerator using a conductive composite hydrogel (PVA/SA@GO CCH) made of poly(vinyl alcohol)/sodium alginate doped with graphene oxide (GO). This device exhibits outstanding mechanical properties, stable electrical output, and broad potential for human applications under various environments [17]. Furthermore, PVA can be repeatedly freeze–thawed to obtain stable hydrogels. The resulting hydrogels exhibit excellent flexibility and anti−fatigue properties [18]. Generally, the more freeze–thaw cycles performed, the higher the crosslink density, resulting in stronger mechanical properties [19]. Moreover, Wu et al. designed a strategy based on the Hofmeister effect to reversibly and extensively modify the mechanical properties of hydrogels, achieving high mechanical properties and broad dynamic tunability in PVA hydrogels, which provides a foundation for their widespread application in biomedicine and wearable devices [20].

Hence, PVA crosslinked with CaCl_2_ was combined with CNTs, and a PVA−Ca−CNT hydrogel was fabricated utilizing a freeze–thaw strategy. CaCl_2_ improves the PVA−Ca−CNT hydrogel with fantastic bonding strength and a Young’s modulus matching that of skin, enabling the PVA−Ca−CNT hydrogel to self−adhere to various substrate surfaces (including metal, inorganic non−metal, polymer substrates, etc.), with a maximum adhesion energy of 305 μJ cm^−2^. The conductive network constructed by CNTs increases the conductivity of PVA−Ca−CNT hydrogel while improving its fracture strain to 648%. As a strain sensor, it has a sensitivity as high as 11.11, which can respond to stimuli within 180 ms and operate stably for more than 35,000 s. PVA−Ca−CNT hydrogel adheres to major joints of the human body and can accurately monitor their movement positions. Moreover, PVA−Ca−CNT hydrogel has demonstrated its accuracy in monitoring minute movements while controlling finger Morse code output. This work provides a new strategy for the investigation of self−adhesive hydrogels in human motion monitoring.

## 2. Experimental Section

### 2.1. Materials

Polyvinyl alcohol (PVA, purity of 97.5–99%) was purchased from Shanghai Mike Biochemical Technology Co., Ltd., Shanghai, China. Multi−walled CNT (length < 10 μm, diameter > 50 nm) dispersion (~10 wt%) was purchased from Xianfeng Nano Co., Ltd. Calcium chloride (CaCl_2_) was purchased from Sinopharm Chemical Reagent Co., Ltd.

### 2.2. Preparation of PVA−Ca−CNT Hydrogel

First, PVA powder (15 g) was dissolved in deionized water (60 mL, 90 °C), and CaCl_2_ solution (40 mL, 750 mg mL^−1^) was added to it. Second, after the PVA had completely dissolved, CNTs (1.5 mL, 100 mg mL^−1^) were added to the solution and mixed thoroughly via mechanical shear stirring (1500 rpm, 2 h). Finally, a freeze–thaw cycling strategy (freezing condition: −40 °C, 8 h; thawing condition: room temperature, 1 h; repeated three times) is utilized to achieve full crosslinking. The composition of each component is shown in Table S1 (Supporting Information).

### 2.3. Self−Adhesion Test of PVA−Ca−CNT Hydrogel

The self−adhesive properties of PVA−Ca−CNT hydrogels were evaluated on a universal testing machine under ambient conditions (25 °C, 50% relative humidity). Five representative substrate materials were selected for testing: artificial skin (medical silicone), PI, borosilicate glass, stainless steel, and plastic. The substrates were secured to the upper jaw using double−sided tape to ensure a flat and clean contact surface. The upper jaw was slowly lowered until the hydrogel and substrate were in full contact under a constant preload of 50 kPa for 60 s, after which the preload was removed. A lap shear test was then conducted at a crosshead speed of 5 mm min^−1^ until complete debonding. During the test, load and displacement were recorded; the nominal shear stress was calculated as the instantaneous force divided by the initial contact area. Each substrate–hydrogel combination was tested on at least five independent samples, and the reported adhesive strength was the average peak stress.

Translated with DeepL.com (free version).

### 2.4. Characterization and Measurements

Microstructures of the materials were characterized with a scanning electron microscope (SEM) (SU8000, Tokyo, JPN). The molecular compositions and phase structures were identified with a Fourier transform infrared spectrometer (FTIR) (Thermo Fisher, Nicolet 6700, USA) and an X−ray diffractometer (XRD) (Bruker Corporation, D8 advance, GER). The tensile and self−adhesion properties of the samples were measured using a universal testing machine (Instron, 3343). The electrical property was tested by using a digital source meter (Keithley 2410).

## 3. Results

### 3.1. Fabrication and Characterization of PVA−Ca−CNT Hydrogels

**Figure 1:**
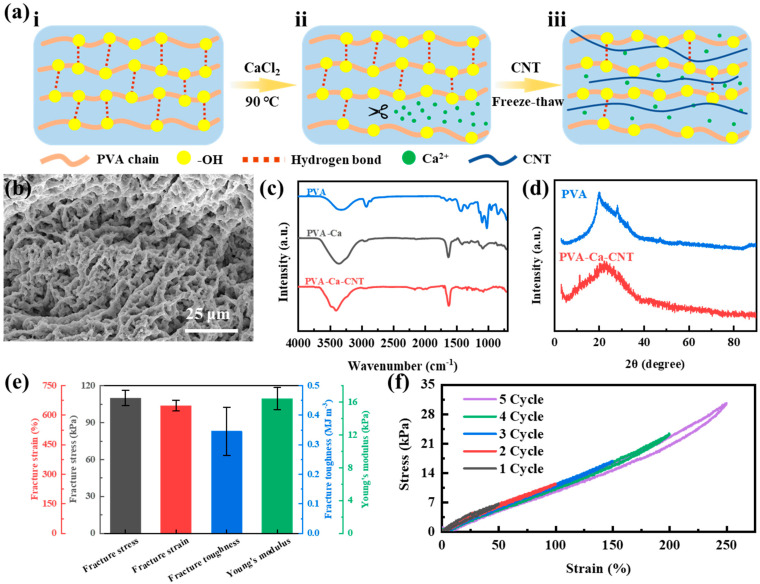
Fabrication and characterization of PVA−Ca−CNT hydrogels. (**a**) Schematic diagram of the fabrication process of PVA−Ca−CNT hydrogel: (i) dissolve PVA, (ii) add CaCl_2_, (iii) add CNT and freeze−thaw. (**b**) SEM image of PVA−Ca−CNT hydrogel. (**c**) FTIR spectra of PVA, PVA−Ca, and PVA−Ca−CNT. (**d**) XRD curves of PVA and PVA−Ca−CNT. (**e**) Mechanical properties of PVA hydrogels. (**f**) Hysteresis curves for PVA hydrogel tensile cycling.

The fabrication schematic diagram of PVA−Ca−CNT hydrogel is shown in Figure 1a. Fabrication of PVA−Ca−CNT hydrogel started with dissolving PVA powder in deionized water at 90 °C to distribute its molecular chains uniformly (Figure 1a(i)). Pure PVA chains, due to the large number of −OH groups, are able to form a large number of hydrogen bonds, resulting in the existence of a substantial crystallization zones. The crystalline zones give the PVA hydrogel a high modulus but also remove its self−adhesive ability and ideal flexibility. To obtain a hydrogel with self−adhesive properties, an adequate amount of CaCl_2_ was added to the PVA aqueous solution (Figure 1a(ii)). The added Ca^2+^ can complex with the −OH groups on the PVA chains to form interchain O−Ca−O interchain crosslinks. At this point, Ca^2+^ shears the interchain hydrogen bonds of PVA like scissors, leading to a decrease in crystallinity in PVA as well as the freeing of more −OH. Notably, Ca^2+^ also opens up a large number of PVA interchain ion channels, allowing excess ions to pass through. To further enhance the conductivity and mechanical properties of PVA−Ca hydrogels, CNTs were added into them (Figure 1a(iii)). The abundant CNTs form a rich conductive network inside the hydrogel (equivalent resistance is 2.749 Ω, Figure S1, Supporting Information), while the CNT network enhances the overall mechanical properties as a mechanical framework. The PVA−Ca−CNT solution was then freeze–thawed several times, water molecules were removed from between the PVA chains, and hydrogen bond crosslinks were formed between the −OH, resulting in a PVA−Ca−CNT hydrogel with self−adhesive ability and ideal modulus. It is worth mentioning that the water content of PVA−Ca−CNT hydrogel is as high as 82.7% (Supplementary Note S1, Supporting Information).

In order to analyze the internal microstructure of PVA−Ca−CNT hydrogels, SEM and EDS images of PVA−Ca−CNT hydrogels were analyzed (Figure 1b and Figure S2, Supporting Information). The three−dimensional interlinked porous microstructure of PVA hydrogel, with rich pores, provides space for the motion of water molecules and ions, and further, the abundant Ca^2+^ (20.8 wt%, Figure S2e, Supporting Information) enhances the overall conductivity, thus significantly enhancing the mechanical properties and conductivity of PVA hydrogel in the stretching process (Table S2, Supporting Information). To further investigate the structural composition of PVA−Ca−CNT hydrogels, the FTIR spectra and XRD curves were analyzed. The wide and strong characteristic peaks at 3200–3600 cm^−1^ for PVA−Ca−CNT, PVA−Ca, and PVA indicate that there are −OH groups from PVA (Figure 1c) [21]. Both PVA−Ca−CNT and PVA−Ca showed significant characteristic stretching vibration peaks from C=C double bonds at 1622 cm^−1^, which are blue−shifted by 22 cm^−1^ relative to the characteristic peak at 1600 cm^−1^ of PVA, indicating that the addition of Ca^2+^ disrupts some of the intra−chain hydrogen bonds (Figure S3a; Supporting Information). Furthermore, the characteristic peak at 3270 cm^−1^ observed in PVA is from stretching vibrations of nonhydrogen−bonded and hydrogen−bonded −OH groups [22]. The absorption peak of the hydroxyl group in PVA−Ca−CNT is at 3403 cm^−1^, blue−shifted by 133 cm^−1^ (Figure S3b, Supporting Information). This is also due to the partial breakdown of the inter−chain hydrogen bonding of PVA by Ca^2+^ [23]. In addition, the absorption peak at 1427 cm^−1^ comes from the bending vibration of −OH. It is worth noting that the characteristic peaks of PVA−Ca−CNT and PVA−CNT are almost identical, indicating that the addition of CNT does not significantly disrupt the formation of intra−chain hydrogen bonds. On the other hand, the PVA crystallization was analyzed using XRD patterns (Figure 1d). PVA has a significant crystallization peak at 20°, which is due to the substantial hydrogen bonding between the PVA chains [20]. With the addition of Ca^2+^, the crystallization peak of PVA−Ca−CNT at 20° tends to become flat, indicating a decrease in the crystallinity. This is caused by Ca^2+^ breaking some of the hydrogen bonds. Moreover, stress–strain tests were conducted on PVA−Ca−CNT hydrogels (Figure 1e and Figure S4, Supporting Information). The PVA−Ca−CNT hydrogel achieved a fracture strain of 648% and a fracture stress of 110 kPa, exhibiting ideal mechanical properties. Furthermore, its Young’s modulus was 16 kPa, consistent with that of human skin, indicating its potential for use as an electronic skin. Additionally, hysteresis properties of PVA−Ca−CNT hydrogels were tested (Figure 1f). For tensile strains of 50%, 100%, 150%, 200%, and 250%, the tensile recovery ratios of PVA−Ca−CNT hydrogel were 95.79%, 95.14%, 94.88%, 93.09%, and 90.12%, respectively. During the recovery process after stretching, the PVA−Ca−CNT hydrogel exhibited virtually no hysteresis loops at the corresponding strains, indicating that the hydrogel’s tensile recovery exhibits minimal hysteresis effects and can maintain stable mechanical properties during cycling.

### 3.2. Self−Adhesive Properties of PVA−Ca−CNT Hydrogels

**Figure 2:**
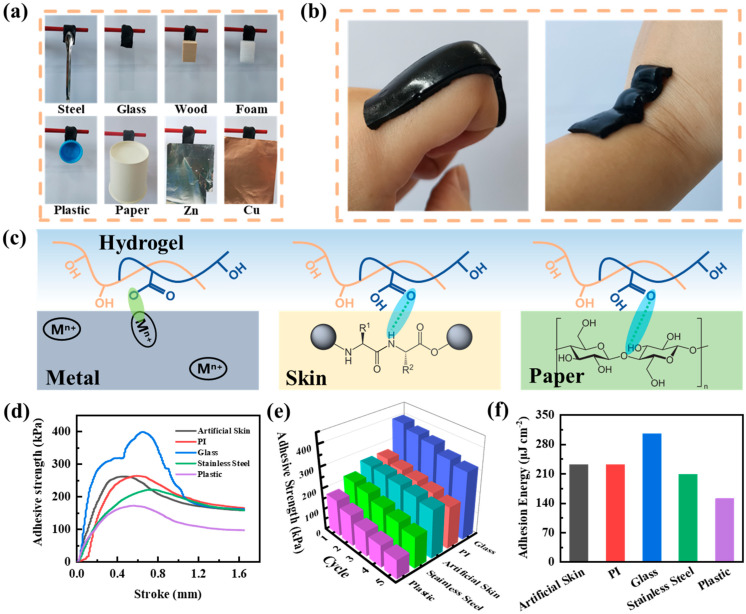
(**a**) Optical images of PVA−Ca−CNT hydrogels adhered on various materials’ surfaces. (**b**) Optical images of PVA−Ca−CNT hydrogel conformal self−adhesion to body’s frequently moving joint surfaces. (**c**) Adhesion mechanism between PVA−Ca−CNT hydrogel and various materials. (**d**) Representative stress–displacement curves for adhesion tests of PVA−Ca−CNT hydrogel to glass, PI, plastic, artificial skin, and stainless steel. (**e**) Adhesion strength and reproducible properties of PVA−Ca−CNT hydrogel adhesion to various materials. (**f**) Area adhesion energy of PVA−Ca−CNT hydrogel adhesion to various materials.

With the strong self−adhesion capability being crucial for the electrode surface to link with the other material’s surface, the self−adhesion property of hydrogels has become one of the most favored properties by researchers. In addition, the self−adhesion capability of hydrogels is essential for the functionalization and practicalization of flexible wearable hydrogels. Benefiting from the abundant hydroxyl and carboxyl groups on the surface of PVA and CNTs, PVA−Ca−CNT hydrogels are able to exhibit universal self−adhesion on a wide variety of materials, for instance, stainless steel, wood, silicone, Ecoflex, and plastics (Figure 2a). In addition, the PVA−Ca−CNT hydrogel closely adhered to the skin at human joints in high−frequency motion with no slipping or peeling, exhibiting strong co−conformability and interfacial adhesion properties (Figure 2b). The self−adhesive ability of PVA−Ca−CNT hydrogels is attributed to the interaction between the large number of functional groups on the hydrogel surface and various material surfaces. Among them, hydroxyl and carboxyl groups can form abundant hydrogen bonds and metal coordination (Figure 2c). In artificial skin, for example, the skin surface is rich in amino and hydroxyl groups, which can form hydrogen bonds with hydroxyl and carboxyl groups of the hydrogel to produce a self−adhesive effect.

To further demonstrate the self−adhesive properties of the PVA−Ca−CNT hydrogel, it was clamped onto various material substrates and subjected to pull−off peeling tests. The maximum value of the peeling curve is taken as the adhesion strength of the PVA−Ca−CNT hydrogel, which indicates the adhesion strength when the adhesion failure occurs, and the corresponding stroke indicates the failure stroke. The adhesion strengths of PVA−Ca−CNT hydrogels on artificial skin, PI, glass, stainless steel, and plastic (all plastics in the manuscript are PE) substrates were 261, 263, 398, 221, and 172 kPa, respectively, which were higher than those of most conductive hydrogels (Figure 2d and Figure S5, Supporting Information). Notably, its failure stroke reaches 0.5, 0.6, 0.66, 0.76, and 0.56 mm, while the failure adhesion force reaches 3922, 3953, 5975, 3324, and 2584 mN, respectively (Figure S6, Supporting Information). It is possible that the cohesive forces existing between PVA and CNTs within the hydrogel dissipate energy during the peeling process, demonstrating ideal tensile strength [24]. The excellent universal self−adhesive properties as well as ideal tensile strength of the PVA−Ca−CNT hydrogel were demonstrated by a pull−off peel test, and it has the potential to be a flexible wearable electrode.

Moreover, PVA−Ca−CNT hydrogels were tested for repeatability of adhesion properties on these substrates. The PVA−Ca−CNT hydrogel was able to retain more than 52% of its original adhesion strength after five pull−off peel tests (Figure 2e; Table S3, Supporting Information). It is attributable to interactions such as reversible hydrogen bonding between the exposed hydroxyl and carboxyl groups on the PVA−Ca−CNT hydrogel surface and the substrate. Remarkably, the PVA−Ca−CNT hydrogel demonstrated strong human adhesion by only decreasing its adhesion strength by 13% after peel–adhesion cycle testing on an artificial skin substrate. In addition, the area adhesion energy between the PVA−Ca−CNT hydrogel and the substrate reaches up to 305 μJ cm^−2^, and, in particular, the area adhesion energy with artificial skin reaches 232 μJ cm^−2^ (Figure 2f). It can be effectively adhered to the human body surface to be used as a flexible wearable electronic device.

### 3.3. Strain Sensing Properties of PVA−Ca−CNT Hydrogels

**Figure 3:**
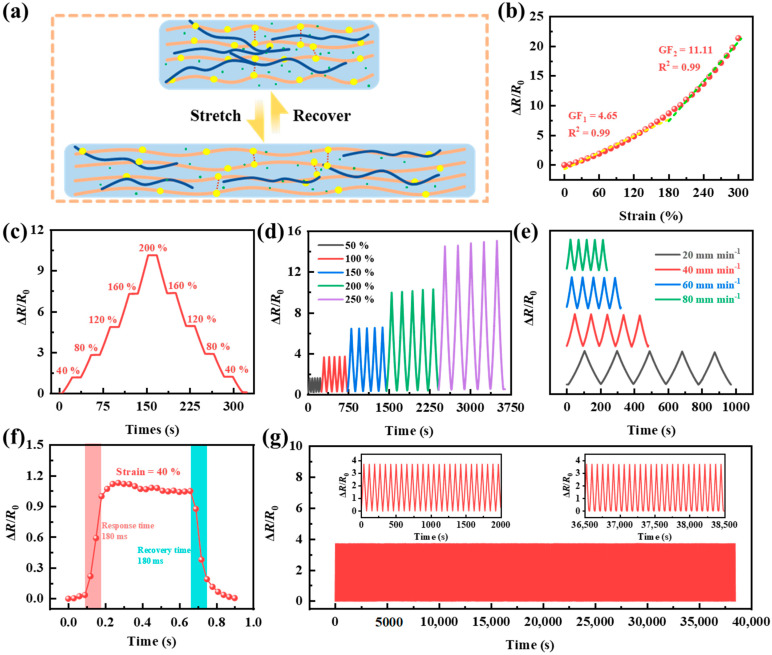
Resistance change ratio of PVA−Ca−CNT hydrogel for strain sensing during stretching. (**a**) Schematic diagram of PVA−Ca−CNT hydrogel for tension sensing. (**b**) Resistance–strain curves of PVA−Ca−CNT hydrogel sensors. (**c**) Changes in resistance change ratio of PVA−Ca−CNT hydrogel strain sensors in stepwise strain stretch and released. (**d**) Resistance change ratio of PVA−Ca−CNT hydrogel at various strains. (**e**) Changes of resistance change ratios with 100% strain under cyclic loading–unloading at various tensile rates (20 to 80 mm min^−1^). (**f**) Response time and recovery time at 40% strain. (**g**) Load–unload cycling curves of PVA−Ca−CNT hydrogels under 100% strain.

Flexible wearable hydrogel sensors are not only required to have self−adhesive properties, but tensile sensing properties are also equally critical. When the PVA−Ca−CNT hydrogel is in an initial state, the inner CNTs are effectively connected together to form a conductive network. At the same time, the Ca^2+^ channel inside the hydrogel is in the normal connectivity state, which can ensure that the conductive ions pass through normally (Figure 3a). When the PVA−Ca−CNT hydrogel was in a tension state under loading, the connections between the CNTs were disrupted and could not form an effective conductive network, which resulted in an increase in resistance. At the same time, the Ca^2+^ ion channels inside the PVA−Ca−CNT hydrogel were partially destroyed, and the conductive ions could not pass through efficiently, resulting in a decrease in the conductivity. In addition, the PVA−Ca−CNT hydrogel exhibits a significant increase in length and decrease in cross−section during tension, which further leads to an increase in the overall resistance of the PVA−Ca−CNT hydrogel. To further investigate the properties of the PVA−Ca−CNT hydrogel sensor, it was clamped on a universal testing machine for tensile testing, while its resistance change was measured using a digital multi−meter. The sensitivity of the PVA−Ca−CNT hydrogel was defined as the gauge factor (GF), calculated from the ratio of the resistance change ratio (Δ*R*/*R*_0_) to the strain value (*ε*). The resistance change ratio of the PVA hydrogel increased with increasing strain and displayed two different sensitivities, GF_1_ and GF_2_ (Figure 3b). The resistance change ratio of the PVA−Ca−CNT hydrogel increased from 0 to 7.3 with stretching within a strain range of 0–160%, demonstrating a sensitivity of 4.65 (GF_1_ = 4.65, R_1_ = 0.99). It is possible there are only changes in the Ca^2+^ ion channels inside the hydrogel at the beginning of the tensile stage, resulting in a slower change in resistance. Over the 160–300% strain range, the PVA−Ca−CNT hydrogel demonstrated a 11.11 sensitivity (GF_2_ = 11.11, R_2_ = 0.99). This is due to the fact that not only are the Ca^2+^ channels disrupted at the late stretching stage but the conductive network composed of inner CNTs is also disrupted, leading to an accelerated increase in the resistance of the hydrogel, demonstrating higher sensitivity.

The PVA−Ca−CNT hydrogel sensors were loaded with stepped strains from 0 to 200% and each strain step was held for 15 s (Figure 3c). It can be seen that the resistance change ratio for strain increasing from 0 to 200% corresponds to the resistance change ratio for strain decreasing from 200% to 0. Particularly, the resistance change ratio is also in a smooth state in the strain−holding phase, showing the stability of the change and the consistency of the resistance change of the PVA−Ca−CNT hydrogel. PVA−Ca−CNT hydrogels underwent dynamic tensile tests, changing their strain levels (from 50% to 250%) with tensile cycles (Figure 3d). The maximum value of the resistance change ratio remains almost constant at each strain level, showing a good dynamic response of the resistance. And the PVA−Ca−CNT hydrogel also demonstrated excellent dynamic stability at various tension rates (Figure 3e). The response/recovery curve is obtained by rapidly loading 40% strain on the PVA−Ca−CNT hydrogel and then releasing it quickly (Figure 3f). The resistance change ratio of the PVA−Ca−CNT hydrogel can rapidly increase within 180 ms as the strain is increasing and stabilizes as the strain is maintained, and the resistance change ratio can return to the preloading state within 180 ms as the strain is released, which indicates that the PVA−Ca−CNT hydrogel is able to sense the strain in a very short time and has a rapidly responding capability. In addition, to verify the working stability of the PVA−Ca−CNT hydrogel, the PVA−Ca−CNT hydrogel was loaded with 100% strain and subjected to a loading–releasing cycle test over 35,000 s (Figure 3g). The resistance change ratio is shown to be around 3.7 for both the first 2000 s and the last 2000 s, with almost no fluctuation. In addition, after a 3 h human thermal stability test (Figure S7), its adhesion strength and tensile strength remained above 60%. It demonstrates that the PVA−Ca−CNT hydrogel has excellent working stability and can be used in life for a long time (Table S4).

### 3.4. Application of PVA−Ca−CNT Hydrogel Strain Sensors

**Figure 4:**
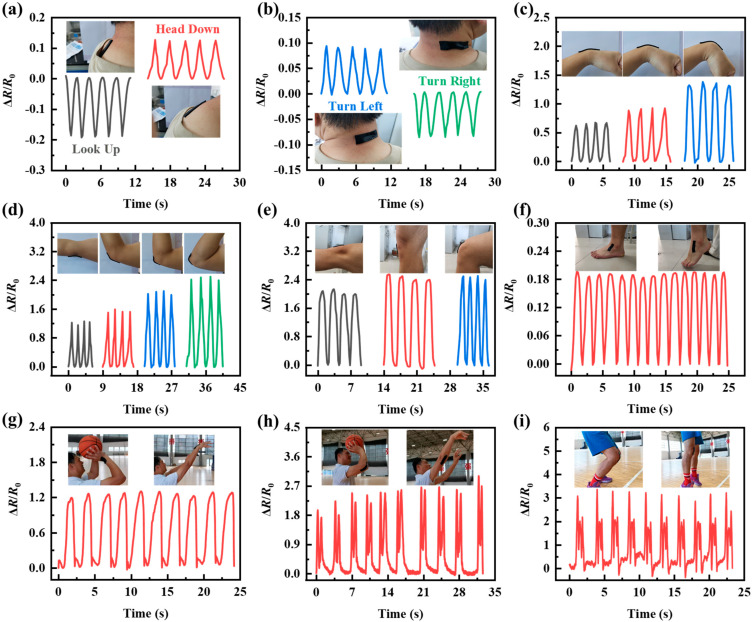
PVA−Ca−CNT−hydrogel−based strain sensors for human motion monitoring. (**a**) Head up and head down. (**b**) Turn head left and right. (**c**) Wrists and (**d**) elbows are bending at different angles. (**e**) Knee bending in different movements. (**f**) Ankle bending. PVA−Ca−CNT−hydrogel−based strain sensors are used to monitor the movement posture of athletes’ (**g**) wrists, (**h**) elbows, and (**i**) knees when shooting.

To demonstrate the applicability and feasibility of PVA−Ca−CNT hydrogel for human motion monitoring, PVA−Ca−CNT hydrogel was adhered to different joints in various parts of the human body to monitor human motion in real time. The PVA−Ca−CNT hydrogel strain sensor adhered to the neck could accurately recognize the movement direction of the head (Figure 4a,b). With prestretching, the PVA−Ca−CNT hydrogel is lengthened and the resistance increases when nodding and turning the head to the left, exhibiting a positive resistance change ratio. When the head is up and turned right, the hydrogel length decreases, the resistance decreases, and this shows a negative resistance change ratio. The PVA−Ca−CNT hydrogel was adhered to the wrist and elbow to monitor the bending motion at different angles (Figure 4c,d). As the bending angle increased (wrist from 30° to 90° and elbow from 30° to 120°), the resistance change ratio of the PVA−Ca−CNT hydrogel increased (from 0.67 to 1.35 with the sensor on the wrist as well as from 1.23 to 2.42 with the sensor on the elbow). And the corresponding maximum value of the resistance change ratio remained almost unchanged during the same angular bending cycle. It is demonstrated that PVA−Ca−CNT hydrogel strain sensors are able to accurately recognize subtle human movement changes. When the PVA−Ca−CNT hydrogel is adhered to the knee’s large joint, the resistance is also able to change with the movement of the knee (Figure 4e). This demonstrates that the PVA−Ca−CNT hydrogel is still able to monitor human joint motion in real time during co−conformal adherence. In addition, the PVA−Ca−CNT hydrogel is adhered to the ankles and is able to monitor the body’s step frequency while walking (Figure 4f). To investigate the specific applications of PVA−Ca−CNT−hydrogel−based strain sensors in daily life, the sensors were attached to an athlete’s wrist, elbow, and knee to monitor their movement posture in real time while playing basketball (Figure 4g–i). Each joint movement can be accurately monitored by strain sensors. In particular, the shaking of the elbow when shooting a basketball and the buffering action of the knee when landing are accurately displayed. This shows that PVA−Ca−CNT−hydrogel−based strain sensors can accurately monitor human joint movement.

**Figure 5:**
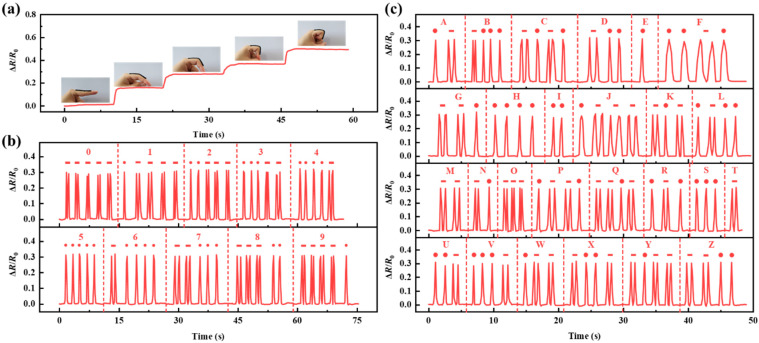
PVA−Ca−CNT hydrogel−based strain sensors for subtle motion recognition. (**a**) Finger bending at different angles. PVA−Ca−CNT hydrogel is adhered to the finger for Morse code signal output of (**b**) Arabic numerals and (**c**) English letters.

Further investigation for the application of PVA−Ca−CNT hydrogel strain sensors on the tiny joints of the human body was carried out, which were adhered to the joints of the index finger and monitored the bending motions at different angles (0°, 30°, 60°, 90°, and 120°; Figure 5a). The resistance change ratio increases as the angle increases and the resistance change ratio curve shows a smooth platform when the bending is stopped. Morse code is an “on” and “off” signaling code which is expressed by arranging letters, numbers, etc. in various output sequences. Specifically, the change in the resistance change ratio of the PVA−Ca−CNT hydrogel was used as the output signal, with a single response labeled as a dot and two continuous responses underlined. The 10 Arabic numerals and 26 letters were recoded as the response signal of the PVA−Ca−CNT hydrogel. It can be seen that the 10 Arabic numerals (Figure 5b) and 26 letters (Figure 5c) can be clearly recognized using the response output of the PVA−Ca−CNT hydrogel strain sensor. This indicates that the monitoring capability of the sensor balances large and subtle motions and can be used in all−round detection of human motion.

## 4. Conclusions

In this work, the interchain hydrogen bonding interactions of PVA were weakened through the complexation effect between CaCl_2_ and PVA, thereby enhancing the self−adhesive properties of PVA, with a maximum self−adhesion strength of 398 kPa and a unit adhesion energy of as high as 305 μJ cm^−2^. Adding CNTs enhances the overall conductivity of the hydrogel while also improving its mechanical properties. The maximum tensile strength of the PVA−Ca−CNT hydrogel is enhanced to 110 kPa, and it has a Young’s modulus of 16 kPa, which matches that of human skin. A cyclic freeze–thaw strategy was utilized to enhance the crosslinking degree of the hydrogel while reducing its hysteresis, resulting in stable operation at 100% strain for over 35,000 s. As a strain sensor, PVA−Ca−CNT hydrogel exhibits sensitivities of 4.65 (0–160%) and 11.11 (160–300%), respectively, as well as ultra−high dynamic stability under various stretching ratios and stretching strengths. Moreover, it exhibits extremely fast response/recovery time (within 180 ms) at 40% strain. As a human motion monitoring sensor, PVA−Ca−CNT hydrogel can accurately recognize the movement positions of major joints in the human body. It can also accurately recognize tiny movements of the fingers and output accurate signals as a finger Morse code generator. The application of PVA PVA−Ca−CNT hydrogel in joint motion monitoring demonstrates its potential in human motion monitoring and intelligent healthcare.

## Data Availability

The original contributions presented in the study are included in the article/supplementary material, further inquiries can be directed to the corresponding authors.

## Supplementary Materials

The following supporting information can be downloaded at: https://www.mdpi.com/article/10.3390/polym17162249/s1, Figure S1. Equivalent resistance of PVA−Ca−CNT hydrogel; Figure S2. EDS images of (a) Ca, (b) Cl, (c) O, and (d) C in PVA−Ca−CNT hydrogel. (e) Elemental proportions in PVA−Ca−CNT hydrogels. Figure S3. Partial FTIR spectrum of PVA, PVA−Ca, and PVA−Ca−CNT. Figure S4. Stress–strain curve of PVA−Ca−CNT hydrogel. Figure S5. Maximum adhesion strength for adhesion test of PVA−Ca−CNT hydrogel to glass, PI, plastic, artificial skin, and stainless steel. Figure S6. Representative force–displacement curves for adhesion tests of PVA−Ca−CNT hydrogels to glass, PI, plastic, artificial skin, and stainless steel. Figure S7. The adhesive strength and tensile strength of PVA−Ca−CNT hydrogel were tested for 3 h of human thermal stability. Table S1. Composition of PVA−Ca−CNT hydrogel. Table S2. Adhesion strength and tensile strength of PVA−Ca−CNT hydrogels at different CaCl_2_ concentrations. Table S3. Adhesion strength and reproducible properties of PVA−Ca−CNT hydrogel adhesion to various materials. Table S4. Comparison of PVA−Ca−CNT hydrogel strain sensors in this work with previously reported hydrogel sensors. Supplementary Note S1. Calculation of water content for PVA−Ca−CNT hydrogel.
