# Supplementary material for: Poly(Vinyl Alcohol)–Carbon Nanotube Self−Adhesive Hydrogels for Wearable Strain Sensors
Source: Polymers (Basel). 2025 Aug 20;17(16):2249. doi: 10.3390/polym17162249 (PMC12389791; doi:10.3390/polym17162249)
Supplement: Supplementary file 1 [file polymers-17-02249-s001.zip › polymers-3718118-supplementary.pdf]

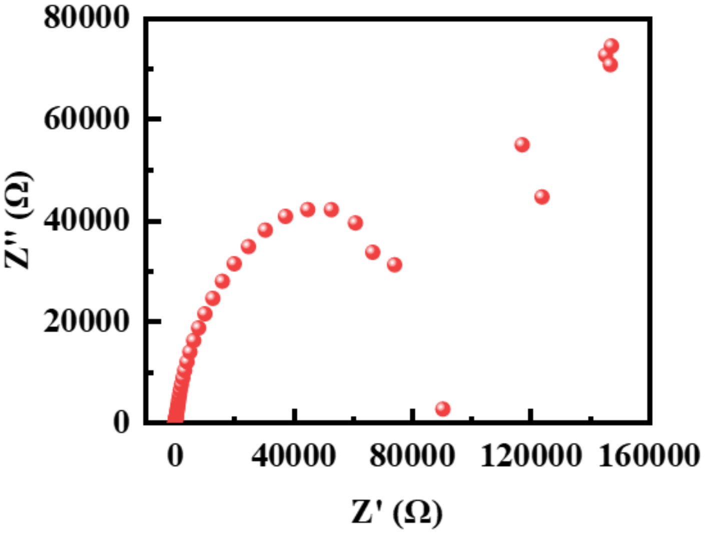

Figure S1. Equivalent resistance of PVA-Ca-CNT hydrogel.

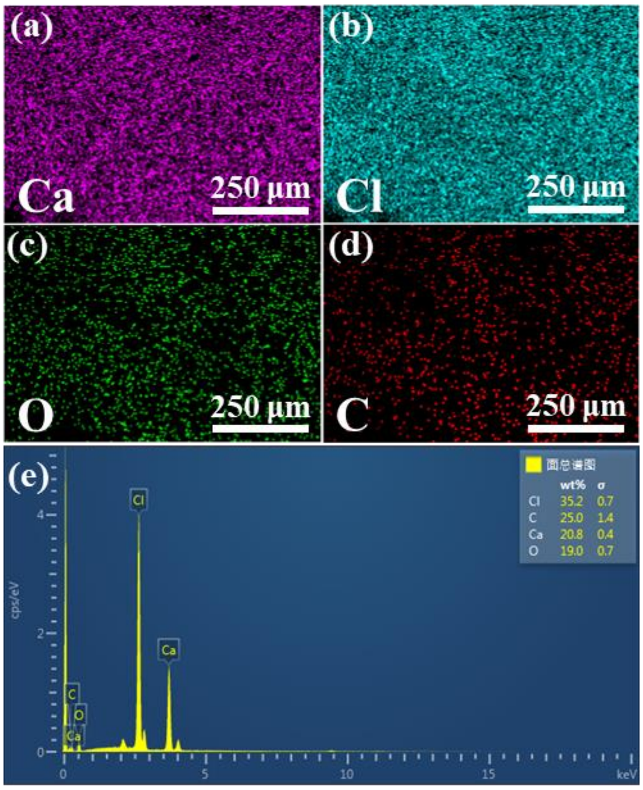

Figure S2. EDS images of (a) Ca, (b) Cl, (c)O, and (d) C in PVA-Ca-CNT hydrogel. (e) Elemental proportions in PVA-Ca-CNT hydrogels.

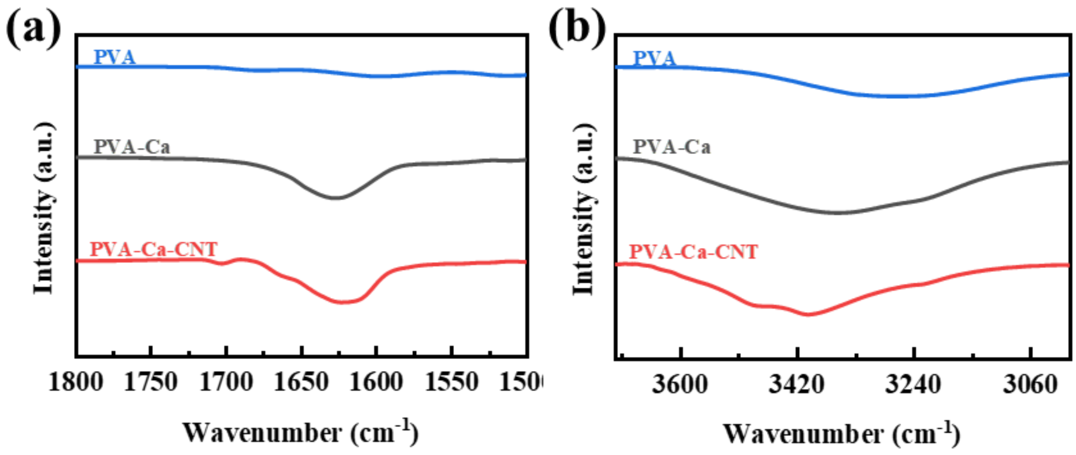

Figure S3. Partial FTIR spectrum of PVA, PVA-Ca and PVA-Ca-CNT.

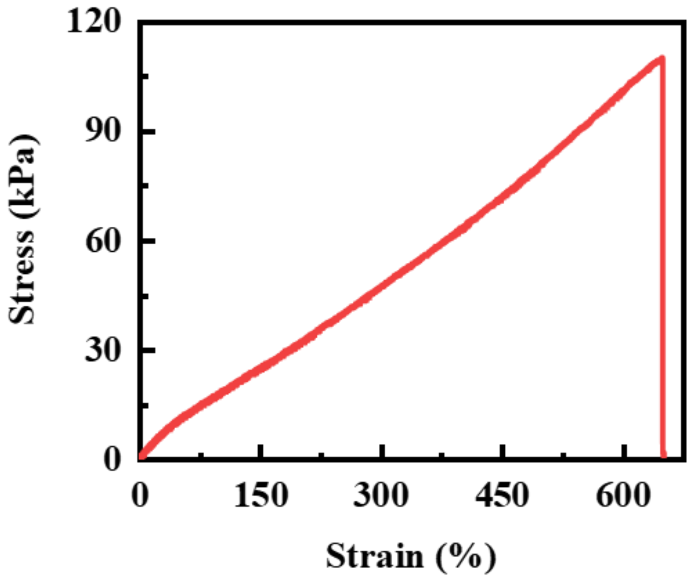

Figure S4. Stress-strain curve of PVA-Ca-CNT hydrogel.

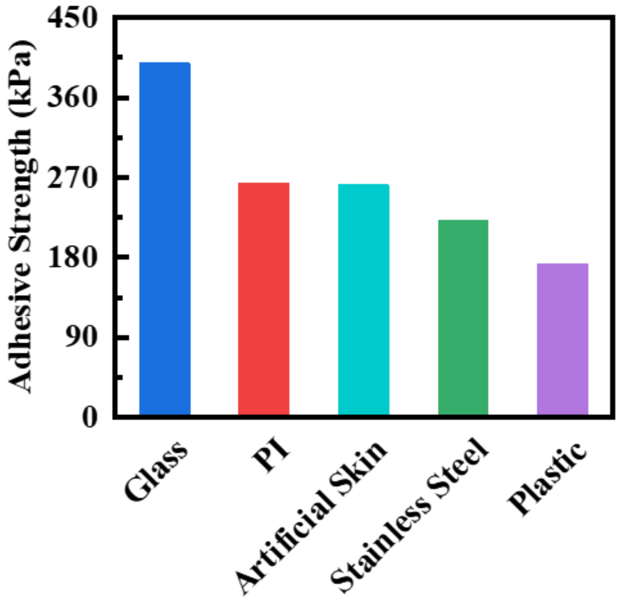

Figure S5. Maximum adhesion strength for adhesion test of PVA-Ca-CNT hydrogel to glass, PI, plastic, artificial skin and stainless steel.

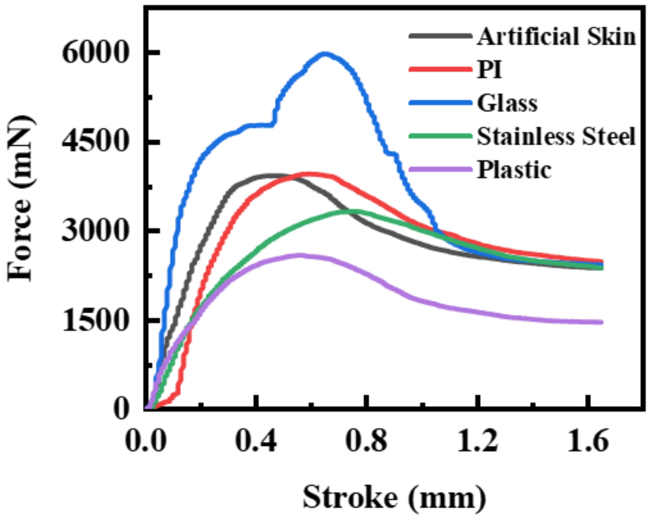

Figure S6. Representative force-displacement curves for adhesion tests of PVA-Ca-CNT hydrogels to glass, PI, plastic, artificial skin and stainless steel.

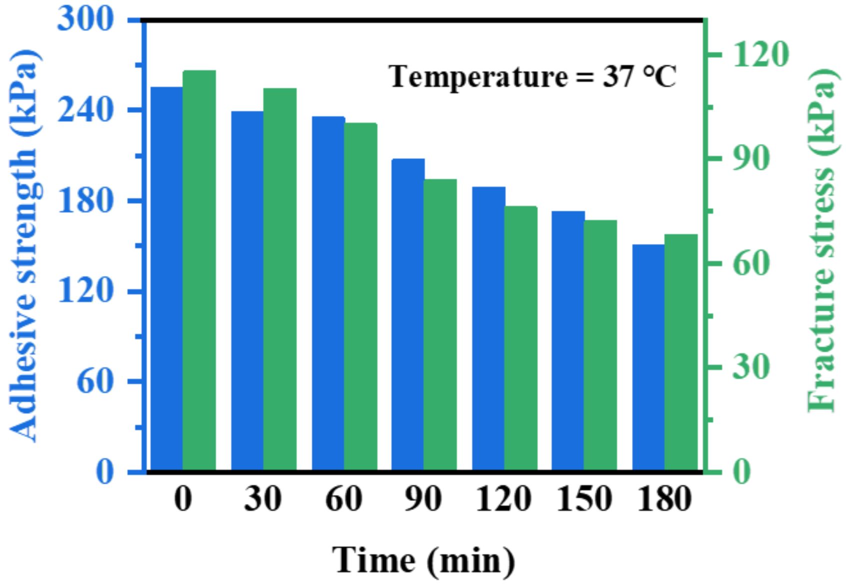

Figure S7. The adhesive strength and tensile strength of PVA-Ca-CNT hydrogel were tested for 3 hours of human thermal stability.

Table S1. Composition of PVA-Ca-CNT hydrogel.

Materials	content
**PVA**	15 wt%
**CNT**	0.15 wt%
**$CaCl_2$**	30 wt%

Table S2. Adhesion strength and tensile strength of PVA-Ca-CNT hydrogels at different $CaCl_2$ concentrations.

$CaCl_2$ concentration	Adhesive strength (kPa)	Tensile strength (kPa)
**20 wt%**	/	150
**30 wt%**	261	110
**40 wt%**	/	/

Note: "/" means no measurement data.

When the $CaCl_2$ concentration is reduced to 20 wt%, the PVA-Ca-CNT hydrogel does not exhibit self-adhesive properties. When the $CaCl_2$ concentration is increased to 40 wt%, PVA-Ca-CNT cannot effectively cross-link to form a hydrogel. When the $CaCl_2$ concentration is 30 wt%, PVA-Ca-CNT can effectively cross-link to form a hydrogel with self-adhesive properties. Therefore, a $CaCl_2$ concentration of 30 wt% was selected.

Table S3. Adhesion strength and reproducible properties of PVA-Ca-CNT hydrogel adhesion to various materials.

	Adhesive Strength (kPa)				
Cycle	Glass	PI	Artificial Skin	Stainless Steel	Plastic
**1**	398	263	261	221	172
**2**	378	247	259	210	147
**3**	365	233	252	185	119
**4**	334	219	241	166	105
**5**	318	197	227	147	91

Table S4. Comparison of PVA-Ca-CNT hydrogel strain sensors in this work with previously reported hydrogel sensors.

Materials	Gauge factor (GF)	Max. strain (%)	Response time (ms)	Reference
**PVA-Ca-CNT**	**4.65 (0-160 %)**<br>**11.11 (160-300 %)**	**300**	**180**	**This work**
CNT/PDMS	4.1 (0-20%)<br>15.4 (20-100 %)	100	1	Micromachines 2023, 14(6), 1106
XSBR/SSCNT	4.24 (< 170%)<br>25.98 (170–214%)	214	200	Adv. Mater. 34(1), 2107309 (2022)

TA@HAP NWs-PVA(W/EG)	2.84 (< 350%)	350	51	Adv. Funct. Mater. 31(21), 2011176 (2021)
MWCNT/MoO3	46.3 (< 60%)	60	50	ACS Nano 13(9), 10469–10480 (2019)
SGC	4.135 (< 200%)<br>8.015 (200–500%)<br>14.507 (500–1000%)	1000	163.1	Adv. Funct. Mater. 32(22), 2112281 (2022)

Supplementary Note 1. Calculation of water content for PVA-Ca-CNT hydrogel.

The water content (σ) of a hydrogel is defined as the ratio of the mass of free water lost to the total mass. It can be described by the following function:

$$\sigma = \frac{m_{water}}{m_1} \times 100\%;$$

$$m_{water} = m_1 - m_2;$$

where m_{water} is the mass of free water, m_1 is the total mass of the hydrogel, and m_2 is the mass of the hydrogel after freeze-drying.

Therefore, the PVA-Ca-CNT hydrogel was cut into a size of 35 mm × 10 mm × 1.6 mm. After 80 hours of freeze-drying treatment, PVA-Ca-CNT hydrogels were obtained as freeze-dried samples that had lost their free water. The results after weighing are as follows:

$$m_1 = 740\ mg;$$

$$m_2 = 128\ mg;$$

Therefore,

$$\sigma = 82.7\%$$
